# The Combination of Gefitinib With ATRA and ATO Induces Myeloid Differentiation in Acute Promyelocytic Leukemia Resistant Cells

**DOI:** 10.3389/fonc.2021.686445

**Published:** 2021-09-28

**Authors:** Luciana Yamamoto de Almeida, Diego A. Pereira-Martins, Isabel Weinhäuser, César Ortiz, Larissa A. Cândido, Ana Paula Lange, Nayara F. De Abreu, Sílvia E. S. Mendonza, Virgínia M. de Deus Wagatsuma, Mariane C. Do Nascimento, Helder H. Paiva, Raquel M. Alves-Paiva, Camila C. O. M. Bonaldo, Daniele C. Nascimento, José C. Alves-Filho, Priscila S. Scheucher, Ana Sílvia G. Lima, Jan Jacob Schuringa, Emanuele Ammantuna, Tiziana Ottone, Nelida I. Noguera, Cleide L. Araujo, Eduardo M. Rego

**Affiliations:** ^1^Department of Medical Images, Hematology, and Clinical Oncology, University of Sao Paulo at Ribeirao Preto Medical School, Ribeirao Preto, Brazil; ^2^Center for Cell-Based Therapy, University of Sao Paulo, Ribeirao Preto, Brazil; ^3^Department of Experimental Hematology, Cancer Research Center Groningen, University Medical Center Groningen, University of Groningen, Groningen, Netherlands; ^4^Department of Pharmacology, University of Sao Paulo, Ribeirao Preto Medical School, Ribeirao Preto, Brazil; ^5^Department of Biomedicine and Prevention, University of Tor Vergata, Rome, Italy; ^6^Santa Lucia Foundation, I.R.C.C.S., Neuro-Oncohematology, Rome, Italy; ^7^Hematology Division, Laboratórios de Investigação Médica 31 (LIM 31), Faculdade de Medicina, University of Sao Paulo, Sao Paulo, Brazil

**Keywords:** epidermal growth factor receptor (EGFR), erlotinib, gefitinib, all-trans retinoic acid (ATRA), acute promyelocytic leukemia (APL), ATRA-resistance, ATO-resistance, arsenic trioxide (ATO)

## Abstract

In approximately 15% of patients with acute myeloid leukemia (AML), total and phosphorylated EGFR proteins have been reported to be increased compared to healthy CD34^+^ samples. However, it is unclear if this subset of patients would benefit from EGFR signaling pharmacological inhibition. Pre-clinical studies on AML cells provided evidence on the pro-differentiation benefits of EGFR inhibitors when combined with ATRA or ATO *in vitro*. Despite the success of ATRA and ATO in the treatment of patients with acute promyelocytic leukemia (APL), therapy-associated resistance is observed in 5-10% of the cases, pointing to a clear need for new therapeutic strategies for those patients. In this context, the functional role of EGFR tyrosine-kinase inhibitors has never been evaluated in APL. Here, we investigated the EGFR pathway in primary samples along with functional *in vitro* and *in vivo* studies using several APL models. We observed that total and phosphorylated EGFR (Tyr992) was expressed in 28% and 19% of blast cells from APL patients, respectively, but not in healthy CD34^+^ samples. Interestingly, the expression of the EGF was lower in APL plasma samples than in healthy controls. The EGFR ligand AREG was detected in 29% of APL patients at diagnosis, but not in control samples. *In vitro*, treatment with the EGFR inhibitor gefitinib (ZD1839) reduced cell proliferation and survival of NB4 (ATRA-sensitive) and NB4-R2 (ATRA-resistant) cells. Moreover, the combination of gefitinib with ATRA and ATO promoted myeloid cell differentiation in ATRA- and ATO-resistant APL cells. *In vivo*, the combination of gefitinib and ATRA prolonged survival compared to gefitinib- or vehicle-treated leukemic mice in a syngeneic transplantation model, while the gain in survival did not reach statistical difference compared to treatment with ATRA alone. Our results suggest that gefitinib is a potential adjuvant agent that can mitigate ATRA and ATO resistance in APL cells. Therefore, our data indicate that repurposing FDA-approved tyrosine-kinase inhibitors could provide new perspectives into combination therapy to overcome drug resistance in APL patients.

## Introduction

The clinical introduction of *all-trans* retinoic acid (ATRA) and arsenic trioxide (ATO) revolutionized the treatment of acute promyelocytic leukemia (APL), leading to a disease-free survival rate of 80-90% (1). Nevertheless, 5-10% of APL patients still relapse due to ATRA or ATO resistance (2). Despite the cytotoxic activities of ATRA and ATO in APL cells, low doses of those agents result in induction of terminal myeloid cell differentiation (3, 4). In this context, previous reports demonstrated that inhibitors of the epidermal growth factor receptor (EGFR) increased ATRA and ATO-induced expression of the myeloid differentiation marker CD11b in AML cells (3–7). Nonetheless, the use of EGFR inhibitors in combination with standard therapy was not previously explored in APL cells resistant to ATRA and ATO.

Non-small cell lung cancer (NSCLC) demonstrated constitutive activation of the epidermal growth factor (EGF)/EGFR pathway, due to mutations on the *EGFR* (8). Although *EGFR* mutations are rare in AML (9–11), the level of EGF—the main EGFR ligand—was elevated in the urine of patients diagnosed with APL and decreased after ATRA-induced complete remission (12). Hence, it is conceivable that the activation of the EGF/EGFR signaling pathway could also confer APL leukemic cells with a survival advantage. However, the prevalence and clinical significance of EGFR and its interactors in APL patients remains unknown.

It has been well established that the distinct dimer interfaces formed between the extracellular domain of the EGF receptor and its respective ligands EGF and amphiregulin (AREG) differentially activate intracellular signaling cascades to regulate cell proliferation and differentiation (13). The EGFR tyrosine kinase inhibitors gefitinib (ZD1839) and erlotinib (CP-358774) are small-molecule compounds that prevent the binding of ATP to the intracellular domain of EGFR, thus impairing autophosphorylation and downstream signal transduction (14). The efficacy and safety of gefitinib and erlotinib as first-line therapies for NSCLC have been demonstrated in several clinical trials and retrospective studies (15). Although there is evidence of patients with co-occurrence of acute myeloid leukemia (AML) and NSCLC, which achieved complete hematological remission when treated with erlotinib monotherapy (16, 17), subsequent studies evaluating the response of AML patients to EGFR inhibitors alone could not corroborate these findings (18–20). In a phase II trial, 26/29 (90%) patients with refractory or relapsed AML who received erlotinib monotherapy discontinued treatment because of disease progression. Nevertheless, combination therapies between differentiation agents with EGFR inhibitors have not been evaluated in AML patients (18).

Here, we evaluated the effects of EGFR pharmacological inhibition in distinct APL models. Gefitinib monotherapy induced apoptosis and inhibited the proliferation of NB4 (ATRA-sensitive) and NB4-R2 (ATRA-resistant) APL cells. Additionally, the combination between gefitinib with ATRA and ATO rewired NB4-R2 and NB4 ATOr (ATO-resistant) cells into sensitivity to standard therapy for APL. *In vivo*, APL mice treated with ATRA alone or in combination with gefitinib exhibited increased overall survival in comparison with the vehicle-treated group.

## Material and Methods

### Chemicals

Gefitinib (#S1025) and erlotinib (#S7786) were purchased from Selleck Chemicals (Houston, TX, USA). ATRA and ATO were purchased from Sigma-Aldrich (St. Louis, MO, USA). Gefitinib, erlotinib, and ATRA were dissolved in dimethyl sulfoxide (DMSO). ATO was dissolved in NaOH (1 M). All compounds were stored at −20°C.

### Cell Culture

The human APL cell lines NB4 (ATRA-sensitive), NB4-R2 (ATRA-resistant), NB4 ATOr (ATO-resistant), and NB4 clone 21 (parental line of NB4 ATOr) were cultured in Roswell Park Memorial Institute 1640 medium (Gibco, Rockville, MD, USA) with 2 mM L-glutamine (Invitrogen, Carlsbad, CA, USA) and 10% of fetal bovine serum (FBS; Vitrocell, Campinas, Brazil) at 37°C in a humidified atmosphere of 5% CO_2_. Cell lines were tested and authenticated by STR DNA fingerprinting analysis (Laboratory of Biochemical Genetics, Department of Genetics, Medical School of Ribeirao Preto – University of Sao Paulo).

### Patient Samples

Primary patient APL blasts, healthy CD34^+^ cells, and plasma samples were collected from BM aspirates. Mononuclear cells were isolated by Ficoll density gradient centrifugation (Histopaque-1077; Sigma-Aldrich). CD34^+^ cells were isolated from the BM of healthy volunteers using the CD34 Microbead Kit (#130-046-703; Miltenyi Biotec, Auburn, CA, USA) according to the manufacturer’s instructions. Plasma was obtained by centrifugation (500 g for 10 minutes) of heparinized BM aspirate and stored in aliquots at −80°C until use. BM CD34^+^ cells or plasma samples from healthy donors were used as controls. The study was approved by the local Research Ethics Committee of the Medical School of Ribeirao Preto, University of Sao Paulo, Ribeirao Preto, Sao Paulo, Brazil (Reference: CAAE 05060818.9.0000.5440). All human samples were collected after obtaining written, informed consent from patients according to the recommendations of the Declaration of Helsinki.

### Apoptosis Assay, Determination of 50% Effective Dose (ED_50_) and Combination Index

To evaluate apoptosis, NB4 and NB4-R2 cells were seeded in 24-well plates at a density of 5 × 10^5^/well and treated with ATO (1–4 μM), gefitinib (5–40 μM, alone or in combination with 2 μM of ATO), erlotinib (5–120 μM), or vehicle (DMSO, 0.01%) for 24 h. To detect apoptotic cells, the cells were washed and resuspended in 100 µL binding buffer, 3 µL Annexin V-fluorescein isothiocyanate (BD Biosciences, San Jose, CA, USA), and 3 µL propidium iodide (PI; 50 μg/ml), followed by an incubation in the dark for 20 min. Fluorescence was detected by flow cytometry on a FACSCalibur instrument (Becton Dickinson, San Jose, CA, USA) and analyzed with FlowJo software (Treestar, Ashland, OR, USA). A minimum of 10 000 events was acquired for each sample. The ED_50_ and combination index were calculated using CompuSyn software (CompuSyn, Paramus, NJ, USA); the latter is a quantitative measure of drug interaction, with a value < 1 or > 1 indicating synergism and antagonism, respectively, and a value of 1 indicating an additive effect (21).

### Proliferation Assay

After 24 h of exposure to gefitinib, cells were washed with phosphate-buffered saline (PBS); 4 mL cold 70% ethanol was then added dropwise to the cell pellet while vortexing, followed by storage at −20°C for up to 15 days before staining. The cells were resuspended and washed with staining buffer (PBS with 1% FBS and 0.09% NaN_3_), and 100 μL of cell suspension (1 × 10^7^/ml) was transferred to a tube containing 5 μL of Ki-67-PE antibody (#12-5698-82, clone: SolA15; eBioscience, San Diego, CA, USA) or PE-conjugated IgG1as an isotype control. After incubation for 30 min, cells were washed twice, resuspended in staining buffer, and analyzed by flow cytometry on a FACSCalibur instrument (Becton Dickinson, San Jose, CA, USA) and analyzed with FlowJo software (Treestar, Ashland, OR, USA). A minimum of 10 000 events was acquired for each sample. Positivity is expressed as a percentage of positive cells and mean fluorescence intensity (MFI).

### Differentiation Assay

For *in vitro* experiments, NB4, NB4-R2, NB4-ATOr, and NB4 clone 21 cells were collected 72 h after drug treatment, washed, and resuspended in 100 μL PBS and incubated with CD11b-PE (#347557, clone: D12), CD11c-APC (#559877, clone: B-ly6), CD15 (#562371, clone: 7C3.rMAb), and CD16 (#557758, clone: 3G8) (BD Biosciences). Cells obtained from BM, or the spleen of leukemia model mice were labeled with antibodies against CD11b-PE (#553311, clone: M1/70), CD117-FITC (#561680, clone: 2B8), Gr1-FITC (#551460, clone: 1A8; all from BD Biosciences), then collected and washed and resuspended in PBS. The percentage of positive cells and MFI were determined by flow cytometry.

### Western Blotting

Whole-cell lysates were prepared with extraction buffer (10 mM EDTA, 100 mM Tris, 10 nM Na_4_P_2_O_7_, 100 mM NaF, 10 mM Na_3_VO_4_, 2 mM phenylmethylsulfonyl fluoride, and 1% Triton X-100) followed by centrifugation at 10 000 × g for 20 min at 4°C. Protein concentration was determined with the Bradford assay and 50 μg of lysate was analyzed by sodium dodecyl sulfate-polyacrylamide gel electrophoresis on a 10% polyacrylamide gel. The proteins were transferred to a polyvinylidene difluoride membrane (Amersham Hybond-P; GE Healthcare, Memphis, TN, USA) that was probed with antibodies against total EGFR (#2232, polyclonal, 1:1000) and phosphorylated (p-)EGFR (Tyr992) (#2235, polyclonal, 1:1000) (both from Cell Signaling Technology, Danvers, MA, USA); SYK (#1240, clone: 4D10, 1:1000; Santa Cruz Biotechnology, Santa Cruz, CA, USA); and β-actin (#A5441, clone: AC-15, 1:60 000; Sigma-Aldrich). Protein bands were visualized using SuperSignal West Dura Extended Duration Substrate (Thermo Fisher Scientific, Waltham, MA, USA) and the Gel Doc XR+ system (Bio-Rad, Hercules, CA, USA).

### Enzyme-Linked Immunosorbent Assay

Plasma EGF and AREG concentrations were measured with the Human EGF Quantikine ELISA Kit (#DEGFR0) and Human Amphiregulin Quantikine ELISA Kit (#DAR00; both from R&D Systems, Minneapolis, MN, USA), respectively, according to the manufacturer’s instructions.

### PCR for Genotyping

DNA was isolated using the QIAamp DNA Mini Kit (Qiagen, Germantown, MD, USA), according to the manufacturer’s instructions, and used as the template for PCR. The 25 μL reaction contained 2.5 μL of 5× reaction buffer, 2 μL of 25 mM MgCl_2_, 3 μL of 10 mM dNTP mix, 2 μL of each primer (5 μM), and 0.2 μL GoTaq DNA polymerase (Promega, Madison, WI, USA). A 3 μL volume of diluted DNA sample (300 ng) was used for conventional PCR, and amplified products (20 μL) were visualized by electrophoresis with Tris–acetic acid–EDTA buffer on a 1.2% (w/v) agarose gel stained with ethidium bromide under ultraviolet light. PCR amplification was performed on a GeneAmp PCR System 9700 thermocycler (Applied Biosystems, Foster City, CA, USA) under the following conditions: 94°C for 5 min; 35 cycles of 94°C for 30 s, annealing at the melting temperature for 45 s, and 60°C for 30 s; and 60°C for 7 min. The following forward and reverse primers were used: PML, 5’-TCAAGATGGAGTCTGAGGAGG-3’ and 5’-CTGCTGCTCTGGGTCTCAAT-3’; and β-actin, 5’-TCTTGATAGTTCGCCATGGAT-3’ and 5’-GGTCATCTTTTCACGGTTGG-3’.

### *In Vivo* Experiments

To investigate the *in vivo* effects of the EGFR inhibitors gefitinib or erlotinib as monotherapy or combined with ATRA, we used a syngeneic transplantation mouse model of APL with leukemia cells from human chorionic gonadotropin (hCG)–promyelocytic locus–retinoic acid receptor A (PML–RARA) transgenic mice (B6129 mixed background), as previously described (22, 23). The hCG-PML-RARA mice were kindly donated by Dr. Pier Paolo Pandolfi (Beth Israel Deaconess Medical Center, Harvard University) and maintained at the Laboratory of Experimental Animal Studies (Fundação Hemocentro de Ribeirão Preto–Ribeirão Preto, SP, Brazil).

8 to 12-week-old male wildtype (WT) littermates, weighing approximately 30 g each, were used as transplant recipients after lethal irradiation (7 Gy split into two doses from an X-ray source i.e., two 3.5 Gy doses, 4 h apart - RS200 from Rad Source Technologies, Inc., Georgia, USA). In the next day, the animals were exposed to a dose of 2% isoflurane for 5 min to induce anesthesia, and immediately afterward 4 × 10^6^ viable leukemic blasts from hCG-PML-RARA mice (200 µL in PBS) were injected intravenously using a syringe with a 30-gauge disposable needle (BD Biosciences) through the retro-orbital sinus. After this time, the mice were monitored and assessed for engraftment analysis. The engraftment was confirmed by conventional PCR analysis of the DNA isolated from 100 μL of heparinized peripheral blood samples collected *via* submandibular vein by using a 5-mm lancet (Goldenrod Animal Lancet, Medipoint, Mineola, NY), under 2% isoflurane anesthesia.

This analysis was done once per week until the *PML*–*RARA* fusion gene expression was detected (**Figure 5A**). After molecular engraftment confirmation in peripheral blood samples, mice were randomly (by the physical method of paper sortition) assigned to treatment groups: gefitinib (100 mg/Kg/day; n=7) and vehicle (1:10 solution of DMSO : PBS; n=7) (**Figure S1A**); gefitinib (200 mg/Kg/day; n=5) and vehicle (n=6) (**Figure S1B**); erlotinib (200 mg/Kg/day; n=4) and vehicle (n=4) (**Figure 4B**); gefitinib (200 mg/kg/day; n=7), ATRA (2.5 mg/kg/day; n=7), gefitinib plus ATRA (n=9), or vehicle (n=5) (**Figure 4D**). Gefitinib (100 or 200 mg/kg/day), erlotinib (200 mg/kg/day), ATRA (2.5 mg/kg/day) diluted in 200 μL of PBS, or vehicle, were administered every day in the afternoon hours during the light cycle by intraperitoneal (i.p.) injection, for 15 consecutive days. The dose, drug administration route, and treatment period for EGFR inhibitors and ATRA have been established based on previous studies (24–28). Overall survival of mice was defined as the length of time from the start of treatment until the date of spontaneous death or euthanasia. All animal experiments were performed at the Laboratory of Experimental Animal Studies (Fundação Hemocentro de Ribeirão Preto – Ribeirão Preto, SP, Brazil).

Animal experiment protocol and experimental procedures were approved by the Animal Care and Use Committee of the Medical School of Ribeirao Preto of the University of Sao Paulo (Protocol no. #016/2016) and conformed to the rules and regulations of the National Council for Control of Animal Experimentation of Brazil (CONCEA). This manuscript was written following the ARRIVE reporting guideline for reporting animal research (29). A completed ARRIVE guidelines checklist is included in **Table S2**.

### Statistical Analysis

Significant differences between groups were evaluated with the unpaired t-test or Kruskal–Wallis test, followed by Dunn’s *post hoc* test. Bivariate correlation analysis with Spearman’s test was performed to determine the correlation between BM plasma concentration of EGF or AREG and WBC count at the time of diagnosis. The log-rank test (with Kaplan–Meier curves) was used for overall survival analysis. Statistical analyses were performed using Prism v.7.03 software (GraphPad, La Jolla, CA, USA). The significance level was set as P ≤ 0.05.

## Results

### EGFR Protein Expression Is Only Detected in a Subset of APL Patients.

**Figure 1:**
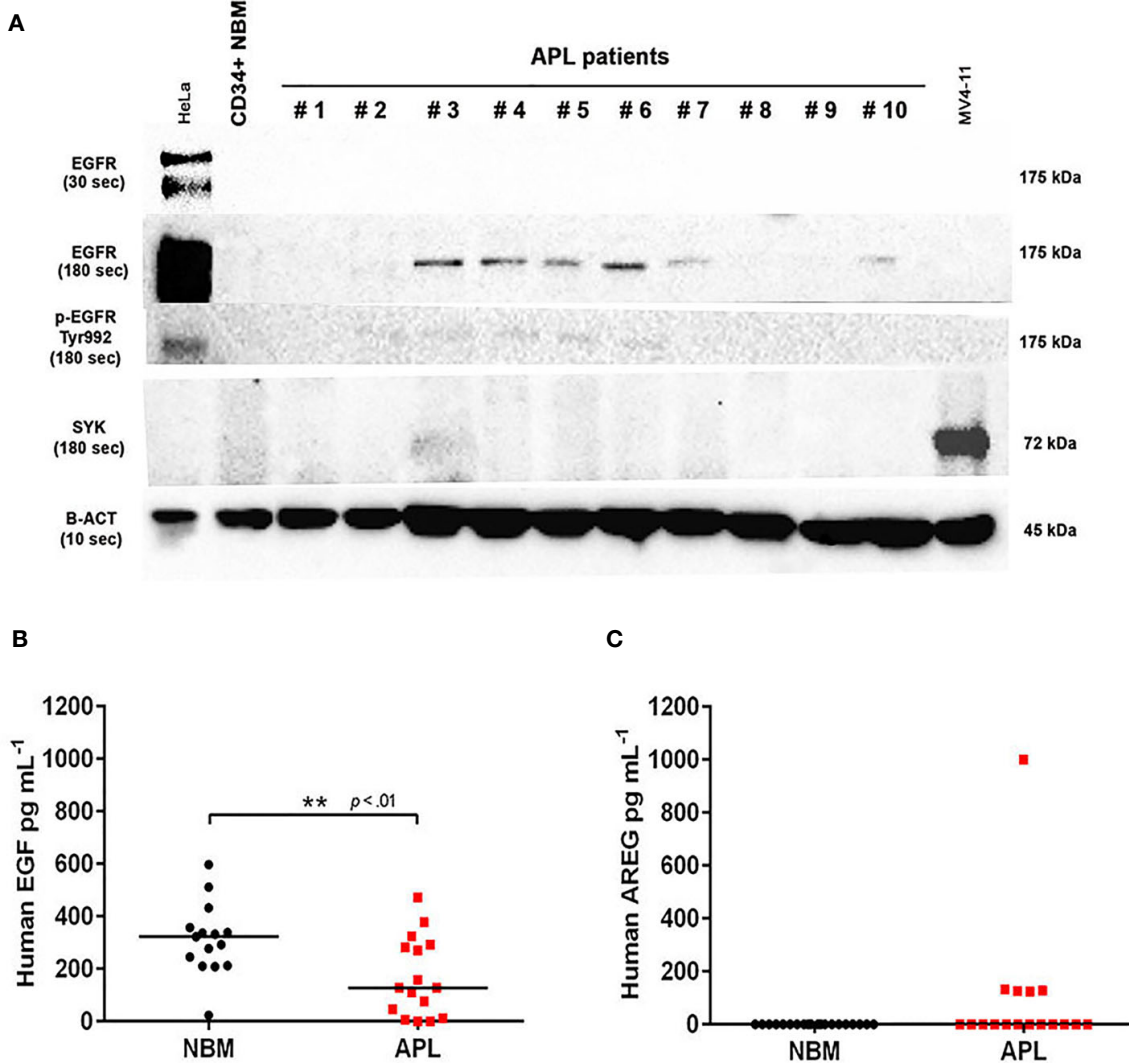
EGFR, p-EGFR (Tyr992), SYK, EGF, and AREG protein expression in APL patient samples. **(A)** Western blotting analysis of EGFR, p-EGFR (Tyr992), SYK, and β-actin protein levels in CD34^+^ cells isolated from normal BM of one healthy adult volunteer and 10 representative primary APL samples collected at diagnosis. HeLa cells served as a positive control to assess EGFR and p-EGFR (Tyr992) expression, and MV4-11 cell extracts were used as a positive control for SYK expression. EGFR and p-EGFR (Tyr992) were detectable when exposed for 180 seconds (sec). **(B)** EGF levels in BM plasma samples from healthy donors (n=15) and APL patients at diagnosis (n=16). **(C)** Plasma AREG levels in BM from healthy donors (n=20) and APL patients at diagnosis (n=17). (**) p < 0.01 (Mann-Whitney U-test).

We evaluated EGFR and the non-receptor tyrosine kinase SYK [a potential off-target of EGFR inhibitors (24)] protein levels in bone marrow (BM) cells obtained from 21 patients diagnosed with APL and a pool of BM-derived CD34^+^ cells isolated from six healthy subjects (controls). EGFR protein was expressed in 6/21 (28.5%) APL patients (**Figure 1A**), but not in control samples. Notably, 4/21 (19%) APL samples also showed positivity for p-EGFR (Tyr992) (**Figure 1A**), indicating activation of the EGF/EGFR signaling pathway. SYK protein expression was neither detected in APL nor control subjects (**Figures 1A** and **S1**). In addition, the *EGFR* gene expression (by real-time quantitative polymerase chain reaction) was not detected in any of these specimens (**Table S1**).

### EGF and AREG Concentrations in BM Plasma Samples

Next, we sought to investigate whether the levels of EGF and AREG (EGFR ligands) measured in the plasma of APL patients correlates with the protein expression of EGFR on APL blasts. The EGF levels were lower in BM aspirates of APL patients at diagnosis (n=16) compared to healthy control subjects (n=15) (median concentration of 127.3 ± 149 *vs* 322.2 ± 136 pg ml^−1^, *P<0.01*) (**Figure 1B**). AREG was detected in BM plasma of 5/17 APL patients at diagnosis but was absent in control samples (n=20) (**Figure 1C**). Among the five AREG-positive BM plasma samples from APL patients, four had a median AREG concentration of 126.8 ± 3.1 pg ml^−1^ (with an outlier sample displaying concentration >1000 pg ml^−1^). No correlation was observed between EGF (n=14) or AREG (n=5) levels in BM plasma of APL patients and peripheral white blood cell (WBC) counts (EGF: r^2^ = 0.01 – **Figure S2A**; AREG: r^2 =^ 0.045 – **Figure S2B**).

### Effects of EGFR Inhibitors Alone or in Combination With ATO or ATRA on APL Cell Lines

**Figure 2:**
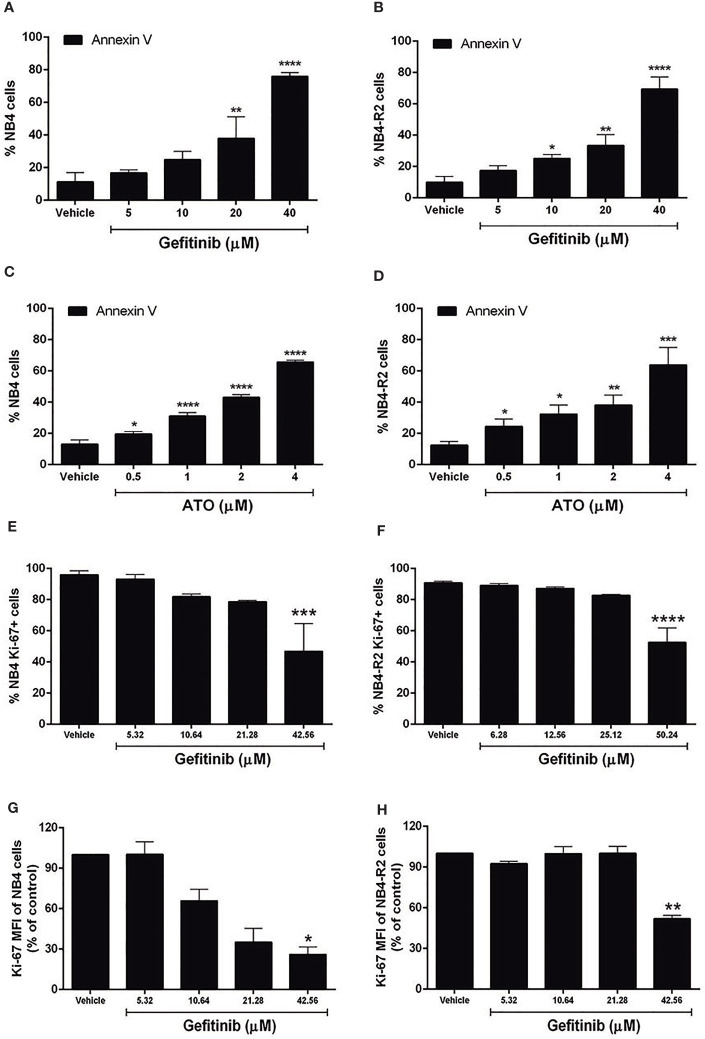
Effects of gefitinib or ATO monotherapy on APL cell apoptosis and proliferation. Gefitinib (5, 10, 20, and 40 μM) **(A, B)** and ATO (0.5, 1, 2, and 4 μM) **(C, D)** treatment for 24 h decreased the fraction of apoptotic NB4 and NB4-R2 cells in a concentration-dependent manner, as determined by Annexin V/PI staining and flow cytometry analysis. Proliferation of NB4 **(E)** and NB4-R2 **(F)** cells was reduced at gefitinib concentrations higher than twice the ED_50_ after 24 h of treatment. **(G, H)** MFI values for NB4 and NB4-R2. Bar graphs show mean ± SD of at least three independent experiments. (*) p < 0.05, (**) p < 0.01, (***) p < 0.001, (****) p < 0.0001 (Kruskal–Wallis test, followed by Dunn’s *post hoc* test).

**Figure 3:**
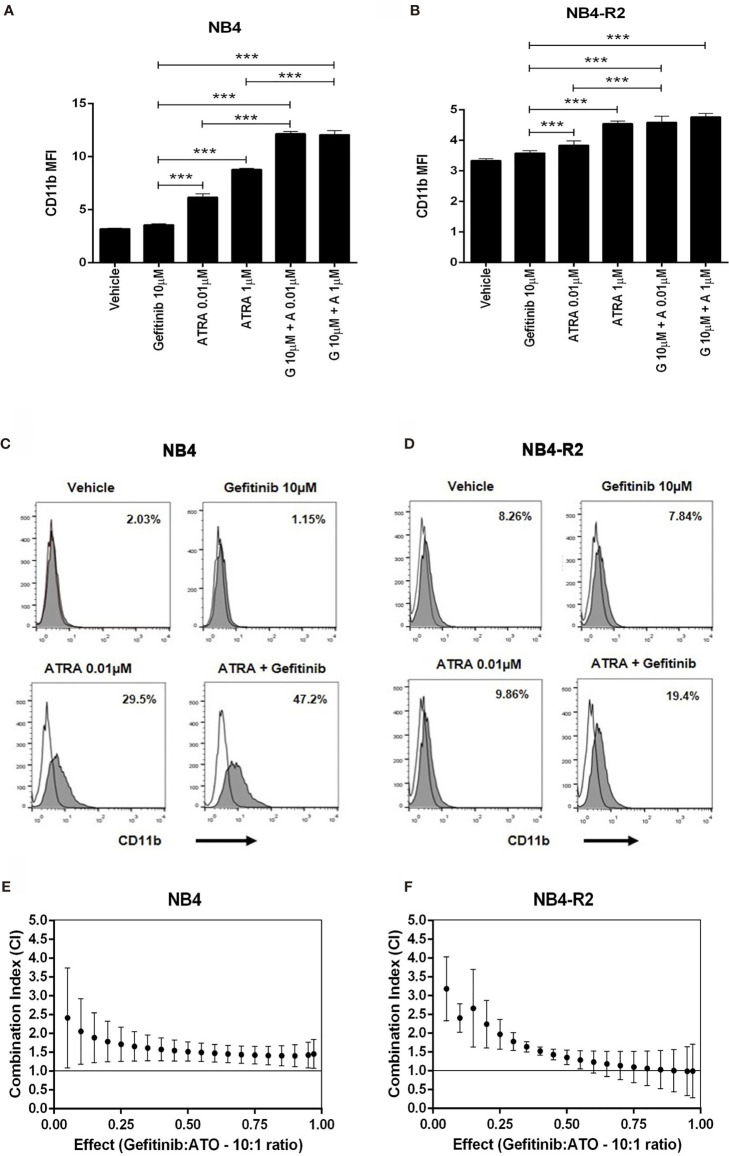
The effect of gefitinib combined with ATRA or ATO on NB4 and NB4- R2 cells. **(A, B)** CD11b MFI of NB4 and NB4-R2 cells treated with ATRA alone (0.01 or 1 μM), gefitinib alone (10 μM), or ATRA plus gefitinib for 72 h. Gefitinib potentiated the myeloid differentiation-inducing effect of ATRA in NB4 **(A)** and NB4-R2 cells **(B)**; representative histograms with percentages for CD11b expression in NB4 **(C)** and NB4-R2 **(D)** cells are shown. Plots show the combination index (CI) versus the fractional effect of gefitinib plus ATO treatment at 10:1 constant ratio in NB4 **(E)** and NB4-R2 **(F)** cells. CI values were calculated by using the CompuSyn software according to the recommendations of Chou and Talalay (21). Data represent the mean ± SD of at least three independent experiments. (***) p < 0.001 (Kruskal– Wallis test, followed by Dunn’s *post hoc* test).

**Figure 4:**
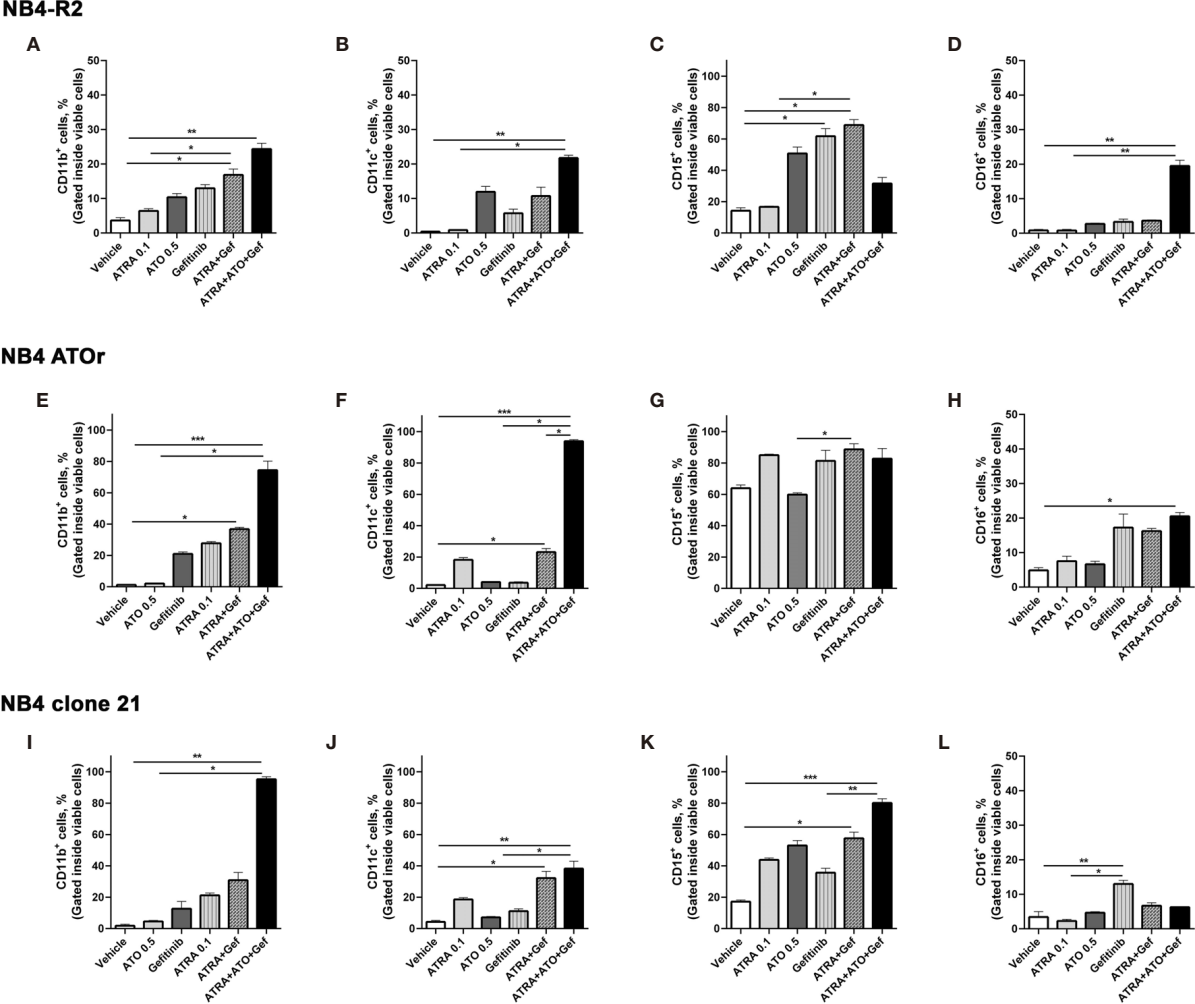
Gefitinib therapy sensitizes APL-resistant cells to ATRA and ATO. The APL cell lines NB4-R2 **(A–D)**, NB4 ATOr **(E–H)**, and the respective parental NB4 clone 21 **(I–L)** were treated with ATRA alone (0.1 µM), ATO alone (0.5 µM), gefitinib alone (10 µM), ATRA plus gefitinib or ATRA and ATO plus gefitinib for 72 h and the expression of the myeloid differentiation markers CD11b, CD11c, CD15, and CD16 was measured by flow cytometry. Gefitinib potentiated the myeloid ATRA-induced differentiation in APL cells resistant to ATRA (NB4-R2) and ATO (NB4 ATOr), as well as in NB4 clone 21 cells. Data represent the mean ± SD of at least three independent experiments. (*) p < 0.05, (**) p < 0.01, (***) p < 0.001 (Kruskal–Wallis test, followed by Dunn’s *post hoc* test).

We first evaluated EGFR and SYK protein levels in NB4 and NB4-R2 cells. As previously demonstrated, both APL cell lines are negative for EGFR (**Figure S3B**) but express SYK (**Figure S3A**), an off-target of EGFR inhibitors. The 50% effective dose (ED_50_) values for the cytotoxic activities of gefitinib, erlotinib, and ATO, as well as the combination index (CI) values for gefitinib plus ATO in the two APL cell lines, are shown in **Table 1**. Since ATRA does not induce cytotoxicity at physiological concentrations after 24h in APL cell lines (30), we did not perform experiments using EGFR inhibitors combined with ATRA. Compared to the vehicle, gefitinib induced increased apoptosis in NB4 cells (**Figure 2A** and **Figure S4A**) and NB4-R2 cells (**Figure 2B** and **Figure S4B**). With a dose-dependent effect, the maximum cell death was observed at 40 μM for gefitinib treatment. Erlotinib had higher ED_50_ values in both NB4 and NB4-R2 cells compared with gefitinib (**Table 1** and **Figure S4C**); therefore, the analysis of synergism with ATO was performed using only the latter (**Table 1** and **Figures 3E, F**). Evaluation of Ki-67 staining (MFI) revealed that gefitinib was only able to impair APL cell proliferation at concentrations > 40 µM in NB4 and NB4-R2 cells (**Figures 2E–H**). In addition, treatment with gefitinib did not alter the proliferative rate of primary human (n=3; **Figure S5A**) or murine (**Figure S5B**) APL blast cells. We next evaluated myeloid differentiation using NB4, NB4-R2, and NB4 ATOr cells (including the respective parental NB4 cells – hereafter called NB4 clone 21) when treated with ATRA (0.01, 0.1 and 1 µM) and ATO (0.5 µM), in the presence or absence of gefitinib (10 µM). Gefitinib monotherapy did not induce APL cell differentiation (**Figures 3A–D**); however, the combined therapy of gefitinib plus ATRA and ATO enhanced APL cell differentiation, measured by the surface expression of the myeloid differentiation markers CD11b, CD11c, CD15, and CD16 (**Figures 4A–L** and **S6**).

**Table 1:** The 50% effective dose (ED_50_) values of gefitinib, erlotinib, and arsenic trioxide (ATO) in NB4 and NB4-R2 cells, and combination index (CI) values of gefitinib plus ATO at different effective levels.

Cell type	ED50^1^			CI^1^(Gefitinib+ATO)			
	Gefitinib(μM)	Erlotinib	ATO(μM)	at ED50	at ED75	at ED90	at ED95
NB4	22.08±5.22	71.66±2.97	2.28±0.14	1.51±0.25	1.42±0.23	1.40±0.29	1.42±0.34
NB4-R2	27.0±7.35	79.95±3.36	2.91±0.94	1.35±0.19	1.10±0.41	1.00±0.56	0.99±0.65

^1^ED_50_ and CI values were calculated by using the CompuSyn software according to the Chou and Talalay method (21).

### Effects of EGFR Inhibitors in an APL Mouse Model

**Figure 5:**
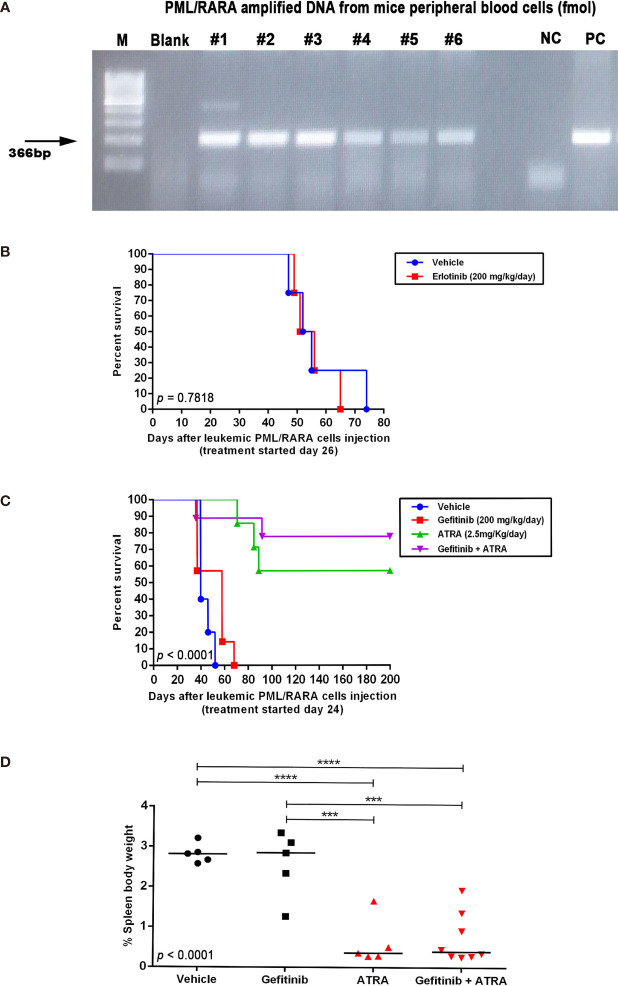
*In vivo* effects of EGFR inhibitors alone or in combination with ATRA in an APL mouse model. **(A)** Expression of the PML–RARA fusion gene in peripheral blood of WT mice 3 weeks after transplantation of leukemic blasts from hCG-PML–RARA transgenic mice detected by conventional PCR. **(B)** Kaplan–Meier survival curves of mice treated with vehicle (DMSO; n=4) or erlotinib at 200 mg/kg/day (n=4). Survival **(C)** and spleen weight-to-body weight ratio **(D)** of mice treated with gefitinib [200 mg/kg/day; n=7 - **(C)** and n=5 - **(D)**], ATRA [2.5 mg/kg/day; n=7 - **(C)** and n=5 - **(D)**], gefitinib plus ATRA [n=9 - **(C)** and n=8 - **(D)**], or vehicle [n=5 - **(C, D)**]. Surviving mice at 200 days post transplantation were sacrificed. (***) p < 0.001, (****) p < 0.0001 (log-rank or Kruskal-Wallis test followed by Dunn’s *post hoc* test).

To investigate the *in vivo* effects of the ATRA/EGFR inhibitor combination, we first assessed the protein levels of Egfr in APL blasts from the transgenic hCG-PML-RARA mice (pool of leukemic blast cells, n=5; **Figure S3B**). No Egfr protein expression was detected in murine APL blasts, suggesting that any potential effects observed upon Egfr monotherapy in this model would be the result of off-target activity. APL transplanted mice were treated for 15 consecutive days with gefitinib (100 or 200 mg/kg/day) or erlotinib (200 mg/kg/day) after confirmation of APL engraftment by PCR analysis of the peripheral blood samples (**Figure 5A**). Mice treated with gefitinib (100 mg/kg/day) (median survival=38 days; 95% confidence interval - CI =34–45 days) (**Figures S7A, B**) or erlotinib monotherapy (200 mg/kg/day; median survival=51 days; 95% CI=44–57 days) (**Figure 5B**) exhibited no prolonged survival compared to the respective vehicle-treated controls (control group for gefitinib: median survival=41 days; 95% CI=35–46 days; control group for erlotinib: median survival=52 days; 95% CI=44–59 days). Treatment with gefitinib at the highest dose (200 mg/kg/day) exhibited a trend to prolong the survival of APL mice (median survival=56 days; 95% CI=47–65 days) (P>0.05).

Due to the increased survival observed in APL mice treated with 200 mg/kg/day of gefitinib, we evaluated if the combination with ATRA (2.5 mg/kg/day) could display additive or synergistic effects *in vivo*. Treatment with ATRA alone or in combination with gefitinib significantly increased the survival rate compared to gefitinib or vehicle (**Figure 5C**), although, there was no difference in survival between mice treated with ATRA alone *versus* ATRA plus gefitinib. Additionally, no differences were observed in spleen weight between the treatment groups (**Figure 5D**). Evaluation of BM and spleen cells regarding the quantification of CD11b^−^CD117^+^ (APL blasts) and CD117^−^CD11b^+^/Gr1^+^ (myeloid mature) cells at the end of the experiment revealed no differences in the frequency of these two leukemic cell populations comparing treatment groups (**Figure S8**). These results suggest that at least *in vivo*, the combination gefitinib plus ATRA did not enhance the differentiation effect of ATRA monotherapy.

## Discussion

In the present study, consistent with previous findings (3, 4) we validated that the combination between gefitinib or erlotinib with ATRA and ATO enhanced the drug-induced myeloid differentiation in APL cells. Moreover, we demonstrate for the first time that this combination was effective for ATRA- and ATO-resistant APL cells, most likely due to an off-target effect. Altogether, our results provide new insights into the ongoing challenge of developing therapies to overcome ATRA and ATO resistance in APL patients.

Despite the anti-leukemic activity of EGFR inhibitors, AML cell lines do not express EGFR (3, 4, 6, 24, 31), implying that gefitinib and erlotinib may act *via* EGFR-independent mechanisms in this malignancy. In this context, other tyrosine kinases have been identified as potential targets of gefitinib and erlotinib, including SYK (32), which predicts a favorable response to fms-like tyrosine kinase 3 (FLT3)-inhibitors in AML patients harboring mutations in the *FLT3* gene (33–35), with no *EGFR* expression. Notably, similarly to ATRA, the inhibition of SYK was reported to induce differentiation of primary APL blasts (32). The NB4 APL cell lines (including the resistant ones), present detectable expression of SYK, although they are negative for EGFR, suggesting that the mechanism underlying the gefitinib-induced APL sensitization to ATRA and ATO might be linked to off-targets downstream the SYK pathway, as demonstrated previously for other AML cell lines (32). Of note, SYK protein expression was not detected in our primary APL samples, raising the possibility of a broader than SYK spectrum of off-target effects upon EGFR inhibitor therapy in APL. Our findings highlight the relevance of repurposing the FDA-approved tyrosine kinase-targeted therapies to overcome the resistance of a specific subgroup of patients with APL who are unresponsive to standard treatment.

Previous *in vitro* studies have shown that differentiation, cell cycle arrest, and apoptosis are induced in AML cells in response to gefitinib and erlotinib, either alone, or in combination with ATRA or ATO (3, 4, 6, 7, 24, 31). Consistent with these findings, we observed that gefitinib enhanced apoptosis and suppressed proliferation in APL cell lines. Mechanistically, the activation of JNK (c-*jun* NH_2_ terminal kinase), a molecular gefitinib off-target (36), plays a crucial role in the ATO-induced apoptosis of APL cells (37), partially explaining the mild cytotoxicity antagonism interaction between ATO and gefitinib.

The EGFR protein was expressed at a low level in 28% of APL BM samples at diagnosis, which was lower than the frequency reported in AML patients (89%). Besides the fact that APL represents a distinct subtype of AML, the difference in EGFR expression could also emanate from different detection methods (Western blot *versus* RPPA). Moreover, p-EGFR (Tyr992) was expressed in 4/6 EGFR-positive samples, suggesting that EGFR activation in APL is similar to other AML subtypes. There was no correlation between *EGFR* mRNA and protein levels, but such disparity has been reported for other genes in different tissues and may reflect post-translational modifications (38, 39). Notably, we found that plasma EGF concentrations were lower in the BM of APL patients compared to healthy controls, in contrast to the higher EGF levels reported in the urine of APL patients at diagnosis (12).

The clinical potential of EGFR inhibitors in AML was first revealed in patients with AML and concurrent NSCLC who achieved complete remission after gefitinib or erlotinib treatment (16, 17, 40). This motivated preclinical studies to assess the efficacy of EGFR inhibitors repurposed into AML clinics. In a mouse xenograft model of AML, erlotinib suppressed tumor growth and increased survival (24). In contrast, in our syngeneic APL mouse model, neither gefitinib nor erlotinib alone induced blast differentiation or prolonged survival. This was consistent with the lack of response observed in patients with advanced AML treated with gefitinib (19). In addition, other studies found that erlotinib monotherapy did not affect cell differentiation or disease remission in AML patients (18, 20). In the present work, the combination of gefitinib and ATRA extended survival in mice and reduced spleen weight-to-body weight ratio compared to gefitinib or vehicle but was not superior to ATRA monotherapy. One limitation of our study is the high sensitivity of hCG-PML/RARA leukemic cells to ATRA monotherapy, in contrast to APL patients, which in the event of relapse frequently show ATRA resistance. Further functional *in vivo* studies are necessary to verify the efficacy of EGFR or other tyrosine kinases inhibitors in APL models resistant to ATRA or ATO treatment.

Although the use of EGFR inhibitors did not prolong survival or increase myeloid differentiation in an APL mouse model sensitive to ATRA, gefitinib stimulated apoptosis, inhibited cell proliferation, and re-sensitized ATRA- and ATO-resistant APL cells to ATRA and ATO induced differentiation, respectively. These findings provide a basis for future studies to explore the potential role of tyrosine kinase-targeted selective therapies in combination with standard therapy, which could be exploited to reverse ATRA and ATO resistance in a subset of patients with APL. Finally, although some of the EGFR signaling components are expressed in APL patient blasts, further investigations are necessary to understand their biological implications on leukemia progression, since the effects of EGFR inhibitors seem to be a result of off-target activities.

## Data Availability Statement

The original contributions presented in the study are included in the article/**Supplementary Material**. Further inquiries can be directed to the corresponding author.

## Ethics Statement

The studies involving human participants were reviewed and approved by Research Ethics Committee of the Medical School of Ribeirao Preto, University of Sao Paulo, Ribeirao Preto, Sao Paulo, Brazil (Reference: CAAE 05060818.9.0000.5440). The patients/participants provided their written informed consent to participate in this study. The animal study was reviewed and approved by Animal Care and Use Committee of the Medical School of Ribeirao Preto of the University of Sao Paulo (Protocol no. #016/2016).

## Author Contributions

*Conceptualization*, ER. *Methodology*, LA, IW, DP-M, CO, LC, APL, NA, SM, VD, MN, JA-F, DN, HP, RA-P, CB, PS, ASGL, CA. *Software*, LA, CB, PS. *Validation*, LA, IW, DP-M, CO, LC, APL. *Formal analysis*, LA, IW, DP-M, CO, ER. *Investigation*, LA, IW. *Resources*, ER. Data curation, LA, IW, DP-M, ER. *Writing—original draft preparation*, LA and ER. *Writing—review and editing*, ER, LA, DP-M, RA-P, JS, EA, TO, NN. *Supervision*, JS, EA, TO, NN. Revision of the manuscript. ER. Project administration, ER, PS, ASGL, CA. *Funding acquisition*, ER. All authors contributed to the article and approved the submitted version.

## Funding

This research was funded by the Fundação de Amparo à Pesquisa do Estado de Sao Paulo (FAPESP; grant no. 2013/08135-2), and FAPESP fellowships for LA (grant no. 2016/02713-2), IW (grant no. 2015/09228-0), DP-M (grant no. 2017/23117-1), CO (grant no. 2017/08430-5), APL (grant no. 2011/17111-4), NA (grant no. 2017/00775-3), VD. (grant no. 2013/11817-8), MN (grant no. 2016/17521-1), HP (grant no. 2010/16966-3), and RA-P (grant no. 2011/18313-0).

## Conflict of Interest

The authors declare that the research was conducted in the absence of any commercial or financial relationships that could be construed as a potential conflict of interest.

## Publisher’s Note

All claims expressed in this article are solely those of the authors and do not necessarily represent those of their affiliated organizations, or those of the publisher, the editors and the reviewers. Any product that may be evaluated in this article, or claim that may be made by its manufacturer, is not guaranteed or endorsed by the publisher.
