# Supplementary figures and images for: The Combination of Gefitinib With ATRA and ATO Induces Myeloid Differentiation in Acute Promyelocytic Leukemia Resistant Cells
Source: Front Oncol. 2021 Sep 28;11:686445. doi: 10.3389/fonc.2021.686445 (PMC8506138; doi:10.3389/fonc.2021.686445)

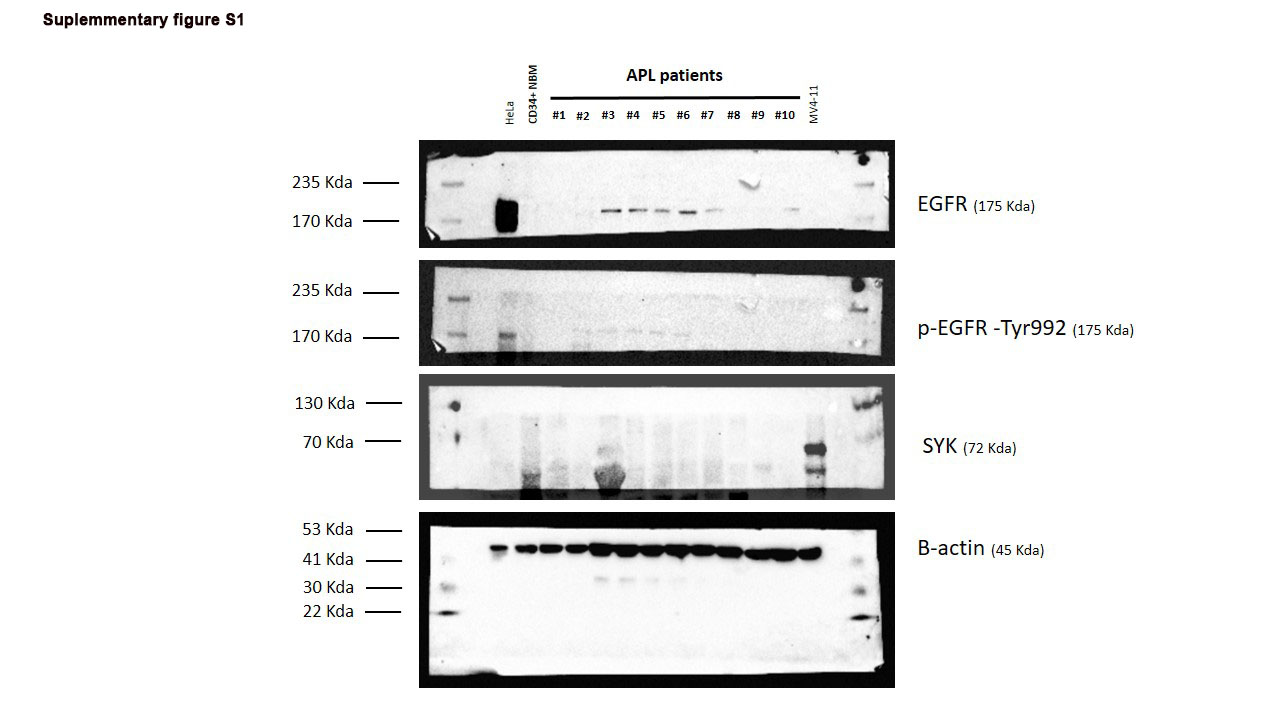

Supplement: Supplementary file 1 [file Image_1.jpeg]

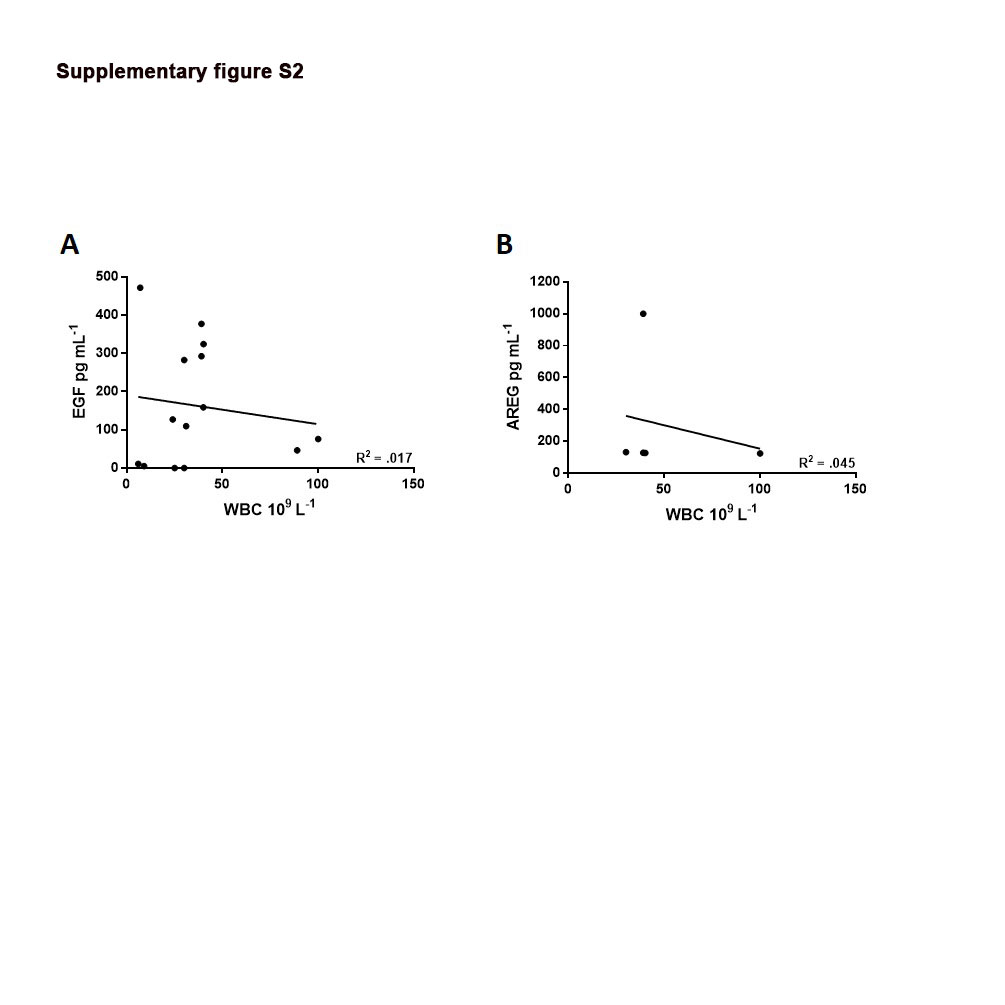

Supplement: Supplementary file 2 [file Image_2.jpeg]

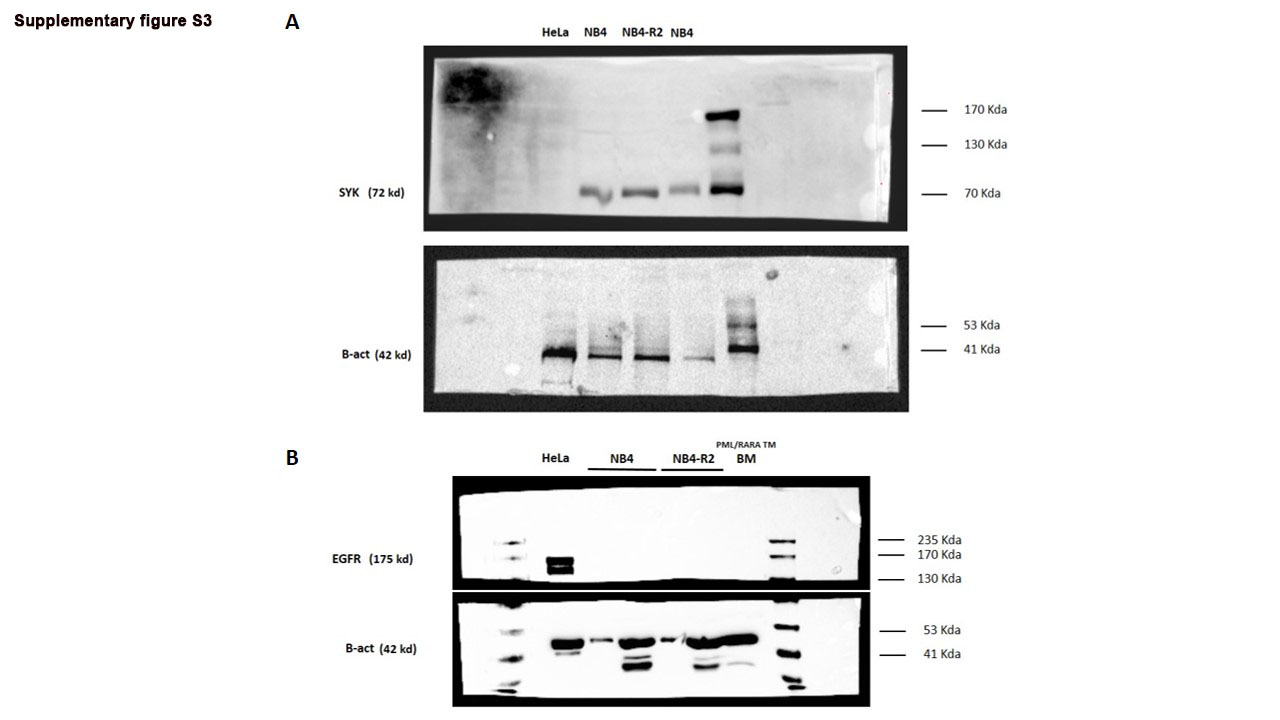

Supplement: Supplementary file 3 [file Image_3.jpeg]

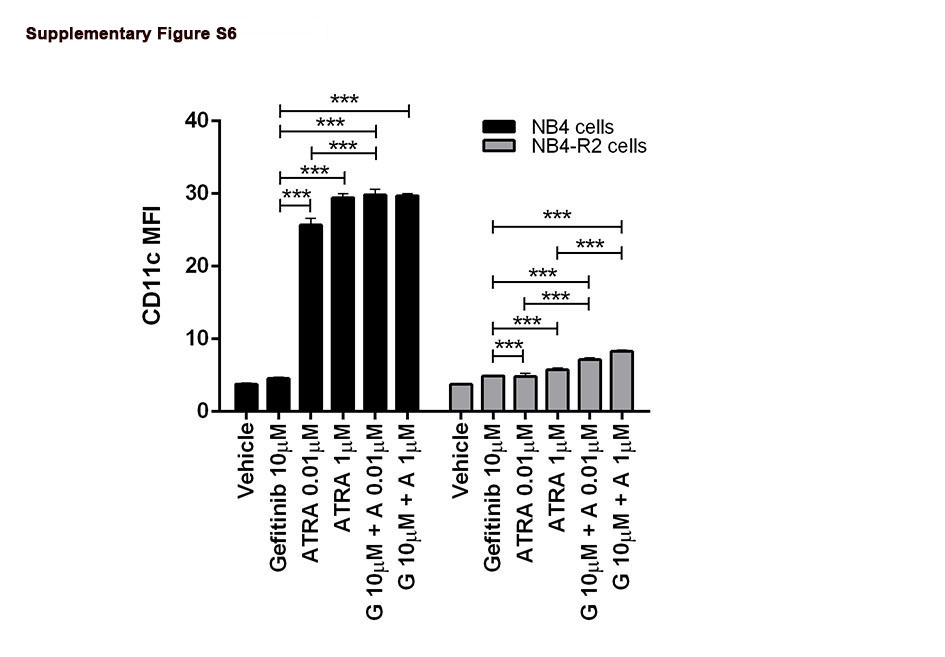

Supplement: Supplementary file 6 [file Image_6.jpeg]

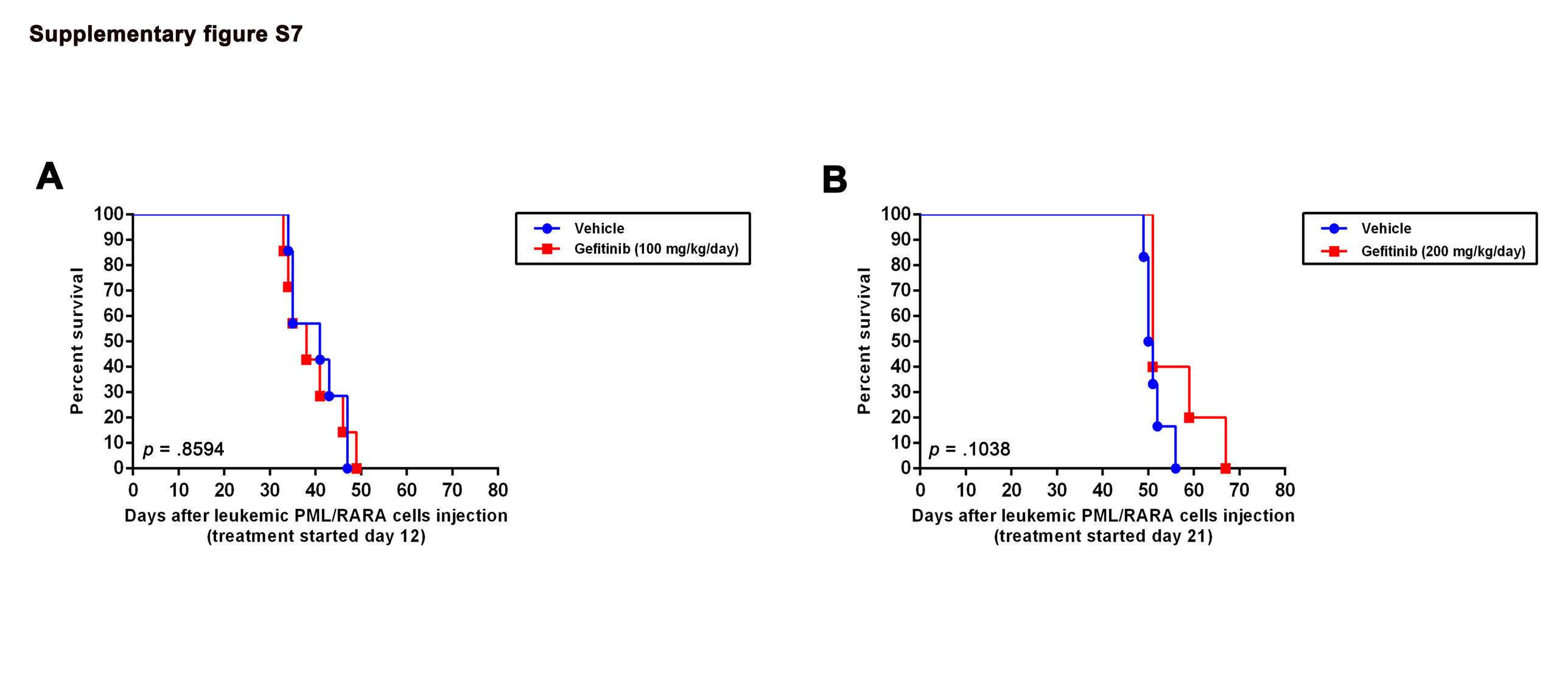

Supplement: Supplementary file 7 [file Image_7.jpeg]

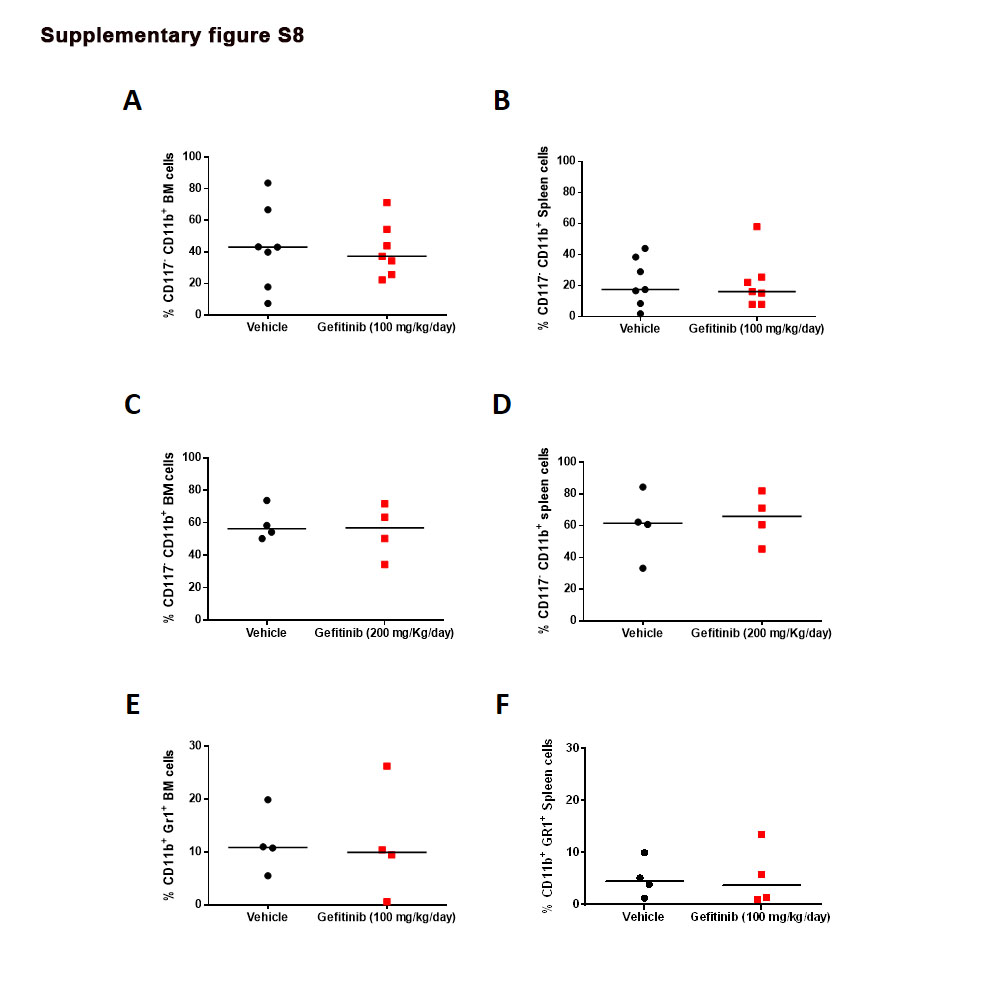

Supplement: Supplementary file 8 [file Image_8.jpeg]
