# Supplementary material for: The Combination of Gefitinib With ATRA and ATO Induces Myeloid Differentiation in Acute Promyelocytic Leukemia Resistant Cells
Source: Front Oncol. 2021 Sep 28;11:686445. doi: 10.3389/fonc.2021.686445 (PMC8506138; doi:10.3389/fonc.2021.686445)
Supplement: Supplementary file 9 [file Table_1.docx]

**Table S1.** Gene expression of *EGFR*

|  | **Mean Ct ^1^** | |
| --- | --- | --- |
| **Sample** | ***EGFR*** | ***GAPDH*** |
| APL 1 | 45.052 | 26.681 |
| APL 2 | Undetermined | 29.323 |
| APL 3 | 41.028 | 32.946 |
| APL 4 | 39.189 | 29.808 |
| APL 5 | Undetermined | 28.214 |
| APL 6 | Undetermined | 25.815 |
| APL 7 | 44.254 | 28.563 |
| APL 8 | 39.358 | 27.587 |
| APL 9 | Undetermined | 27.930 |
| APL 10 | Undetermined | 32.637 |
| HeLa | 27.964 | 30.752 |
| CD34^+^ 1 | Undetermined | 30.823 |
| CD34^+^ 2 | Undetermined | 33.369 |
| CD34^+^ 3 | Undetermined | 24.399 |
| CD34^+^ 4 | Undetermined | 33.942 |
| CD34^+^ 5 | Undetermined | 25.853 |
| CD34^+^ 6 | 39.161 | 21.092 |

^1^ Ct threshold = 0.05
